# Investigation of the Plasma Virome from Cases of Unexplained Febrile Illness in Tanzania from 2013 to 2014: a Comparative Analysis between Unbiased and VirCapSeq-VERT High-Throughput Sequencing Approaches

**DOI:** 10.1128/mSphere.00311-18

**Published:** 2018-08-22

**Authors:** Simon H. Williams, Samuel Cordey, Nishit Bhuva, Florian Laubscher, Mary-Anne Hartley, Noémie Boillat-Blanco, Zainab Mbarack, Josephine Samaka, Tarsis Mlaganile, Komal Jain, Valerie d’Acremont, Laurent Kaiser, W. Ian Lipkin

**Affiliations:** a Center for Infection & Immunity, Columbia University, New York, New York, USA; b Laboratory of Virology, University Hospitals of Geneva, Geneva, Switzerland; c University of Geneva Medical School, Geneva, Switzerland; d Department of Ambulatory Care and Community Medicine, University of Lausanne, Lausanne, Switzerland; e University of Lausanne Medical School, Lausanne, Switzerland; f Swiss Tropical and Public Health Institute, University of Basel, Lausanne, Switzerland; g Infectious Diseases Service, University Hospital of Lausanne, Lausanne, Switzerland; h Mwananyamala Hospital, Dar es Salaam, United Republic of Tanzania; i Ifakara Health Institute, Dar es Salaam, United Republic of Tanzania; Boston University School of Medicine

**Keywords:** UHTS, VirCapSeq-VERT, febrile illness, sequencing, virology

## Abstract

High-throughput sequencing can provide insights into epidemiology and medicine through comprehensive surveys of viral genetic sequences in environmental and clinical samples. Here, we characterize the plasma virome of Tanzanian patients with unexplained febrile illness by using two high-throughput sequencing methods: unbiased sequencing and VirCapSeq-VERT (a positive selection system). Sequences from dengue virus 2, West Nile virus, human immunodeficiency virus type 1, human pegivirus, and Epstein-Barr virus were identified in plasma. Both sequencing strategies recovered nearly complete genomes in samples containing multiple viruses. Whereas VirCapSeq-VERT had better sensitivity, unbiased sequencing provided better coverage of genome termini. Together, these data demonstrate the utility of high-throughput sequencing strategies in outbreak investigations.

**IMPORTANCE** Characterization of the viruses found in the blood of febrile patients provides information pertinent to public health and diagnostic medicine. PCR and culture have historically played an important role in clinical microbiology; however, these methods require a targeted approach and may lack the capacity to identify novel or mixed viral infections. High-throughput sequencing can overcome these constraints. As the cost of running multiple samples continues to decrease, the implementation of high-throughput sequencing for diagnostic purposes is becoming more feasible. Here we present a comparative analysis of findings from an investigation of unexplained febrile illness using two strategies: unbiased high-throughput sequencing and VirCapSeq-VERT, a positive selection high-throughput sequencing system.

## Abstract (precis)

Characterization of the viruses found in the blood of febrile patients provides information pertinent to public health and diagnostic medicine. PCR and culture have historically played an important role in clinical microbiology; however, these methods require a targeted approach and may lack the capacity to identify novel or mixed viral infections. High-throughput sequencing can overcome these constraints. As the cost of running multiple samples continues to decrease, the implementation of high-throughput sequencing for diagnostic purposes is becoming more feasible. Here we present a comparative analysis of findings from an investigation of unexplained febrile illness using two strategies: unbiased high-throughput sequencing and VirCapSeq-VERT, a positive selection high-throughput sequencing system.

## OBSERVATION

High-throughput sequencing (HTS) has transformed outbreak investigation by enabling the rapid identification of known and novel pathogens. Culture remains essential for mechanistic studies, as well as for the development of drugs and vaccines; however, culture is more labor-intensive and may fail for infectious agents with fastidious growth requirements (1). As the cost for data generation through HTS continues to decrease and platforms become smaller and more portable, there is an urgent need for methods that simplify and focus data acquisition and analysis. We have employed two complementary methods to address this challenge in diagnostic virology: (i) a method for positive selection of the viral template prior to sequencing and (ii) a simplified HTS analysis pipeline. We describe the results obtained with these methods using plasma samples obtained from patients diagnosed with febrile illness in Dar es Salaam, Tanzania.

Twelve plasma specimens from patients with unexplained febrile illness were used for comparative analysis. Samples were collected from a larger cohort of adults attending outpatient departments in Dar es Salaam, Tanzania, between August 2013 and April 2014, enrolled in an observational cohort to determine the causes of fever. These 12 patients were considered to be at highest risk of having unidentified viral infections as the cause for the febrile episode. Malaria was excluded as a cause of fever in these patients following screening with a standard malaria rapid diagnostic test (mRDT) (SD Bioline Malaria AG P.f., Standard Diagnostics, Inc., Republic of Korea) that has a limit of detection (LOD) at approximately 50 parasites/µl. This threshold is considered to cover the pyrogenic threshold in adults in areas where malaria is endemic (2). Samples were also screened for low-density parasitemia using an ultrasensitive mRDT (Allere Malaria AG P.f., Standard Diagnostics, Inc., Republic of Korea) (LOD, 5 parasites/µl) and a quantitative PCR (qPCR) targeting the *var* gene acidic terminal sequence (varATS) (LOD, 0.1 parasites/µl) (3–5). All twelve samples were negative for malaria parasitemia by ultrasensitive mRDT and 11 of 12 were negative by qPCR. A single sample (patient 7) was quantified near the LOD at 0.1 parasites/μl. This study was approved by the Ifakara Health Institute Review Board (IHI/IRB number 12-2013) and the Medical Research Coordinating Committee of the National Institute for Medical Research (NIMR/HQ/R.8a/Vol. IX/1561) of Tanzania, and the Ethics Committee of the canton of Basel, Switzerland (reference number EK: 1612/13).

Plasma samples for unbiased HTS were centrifuged at 10,000 × *g* for 10 min. The supernatant was treated with 40 U of Turbo DNase (Ambion, Rotkreuz, Switzerland) prior to RNA and DNA extraction and library preparation (6). RNA libraries for samples 1 to 6 were loaded on a HiSeq 4000 system (Illumina, San Diego, CA, USA) using a multiplex of four samples per lane, while those from samples 7 to 12 were loaded on a HiSeq 2500 system (Illumina, San Diego, CA, USA) using a multiplex of four samples per lane. All DNA libraries were loaded on a HiSeq 4000 system (multiplex of six samples per lane). Raw data were analyzed using an updated version of the ezVIR pipeline (6). FastQ files for each sample were demultiplexed and checked for quality, and adaptors were trimmed using Trimmomatic (v0.33) (7). The resulting files were filtered for low complexity using TagDust (v2.31) (8) and host-subtracted using SNAP (v1.0beta.23) (9). All remaining reads were then aligned to each virus contained in an in-house curated viral database (6) (available at http://unige.ch/virology/home/db/ezvir), also using SNAP (v1.0beta.23) (9). Genome detection metrics, including percent coverage, maximum depth, and total nucleotide coverage are calculated for each detected virus, and results are provided in a two-phase report. The sequence cross talk generated by multiplexing libraries was checked using a 0.3% cutoff (10). PCR confirmation for the presence of West Nile virus (WNV), dengue virus 2 (DV2), and human pegivirus (HPgV) RNA was performed as described in Table 1.

**TABLE 1:** PCR assays used to confirm viruses identified from each sequencing strategy

PCR assay and virus^a^	Target gene^b^	Forward primer or probe (5′→3′)^c^	Reverse primer (5′→3′)^d^	Product size (nt)^e^	AT (°C)^f^	Design or reference^g^
VirCapSeq-VERT						
DV2	NS5	F: AACCTGGTCCATACACGCC	R: CTACCACAAGACTCCTGCC	325	60TD	Designed from HTS data
EBV (qPCR)	EBER	F: AAACCTCAGGACCTACGCTGC	R: AGACACCGTCCTCACCAC	105	60	Modified from reference 20
		Probe: FAM-CCCGTCCCGGGTACAAGTCCC- TAMRA				
HIV-1	*pol*	F1: CAGCAGTACAAATGGCAG	R1: CCACACAATCATCACCTGCC	324	60TD	Designed from HTS data
		F2: GCAGTACAAATGGCAGTTTTC	R2: CACAATCATCACCTGCCATC	319	60TD	
HPgV group 1	NS3	F1: CGTSGTSMTYTGYGACGAGTGCCA	R1: CCRCGCCGCTGCATVCGSAAYGC	533	50	21
		F2: CAYGYDATCTTYTGYCACTCGAAGG	R2: CRAAGTTBCCDGTGTAGCCDGTGGA	177	50	
HPgV group 2	NS3	F1: GSGCNATGGGNCCNTAYATGGA	R1: GTNACYTCVACNACCTCCTCYACCA	615	50	21
		F2: GTGGTNATHTGYGAYGAGTGYCA	R2: TCRCACTCMRCCTTKGARTGRCARAA	265	50	
WNV	NS5	F: TGGATGGAATGTGCGCGACAC	R: TCGTGTGCCAATCAGACTGCC	331	60TD	Designed from HTS data
Unbiased HTS noncommercial						
HPgV (qPCR)	5′ NCR	F: GGCGACCGGCCAAAA	R: CTTAAGACCCACCTATAGTGGCTAAA	93	55	22
		Probe: FAM-TGACCGGGATTTACGACCT ACCAACCCT-TAMRA				
Unbiased HTS commercial^h^						
DV2						
WNV						

^a^ Abbreviations: DV2, dengue virus 2; EBV, Epstein-Barr virus; qPCR, quantitative PCR; HIV-1, human immunodeficiency virus type 1; HPgV, human pegivirus; WNV, West Nile virus.

^b^ NS5, nonstructural protein 5; EBER, EBV-encoded RNA; 5′ NCR, 5′ noncoding region; NS3, nonstructural protein 3.

^c^ Forward primers are indicated by F, F1, or F2 before a colon. FAM, 6-carboxyfluorescein; TAMRA, 6-carboxytetramethylrhodamine.

^d^ Reverse primers are indicated by R, R1, or R2 before a colon.

^e^ nt, nucleotides.

^f^ AT, PCR annealing temperature; TD, touchdown PCR (temperature decreased 0.5°C each cycle for 10 cycles).

^g^ HTS, high-throughput sequencing.

^h^ Tropical fever core real-time reverse transcription-PCR (RT-PCR) from Fast Track Diagnostics (Sliema, Malta).

For VirCapSeq-VERT libraries, total nucleic acid was extracted from plasma using the NucliSens easyMAG automated platform (bioMérieux, Boxtel, The Netherlands). Individual libraries were prepared with the Hyper Prep kit (KAPA Biosystems, Boston, MA, USA) using unique barcodes. Libraries were pooled and hybridized with the VirCapSeq-VERT probe set prior to a final PCR and sequencing on the HiSeq 4000 system using the equivalent of half a lane. Demultiplexed FastQ files were adaptor trimmed using cutadapt (v1.8.3) (11), quality filtered and end trimmed with PRINSEQ software (v0.20.3) (12), and cleaned of host sequences using Bowtie 2 mapper (v2.2.9) (13). Assembly of remaining reads was performed using MIRA Assembler (v4.0) (14). Assembled contiguous sequences and unique singletons were subjected to homology searches against the entire GenBank nucleotide database using MegaBLAST. If sequences showed poor homology, they were subjected to a second round of homology search against the viral GenBank protein database using BLASTX. The filtered reads were mapped with Bowtie 2 to the viral genomes identified from MegaBLAST and BLASTX to determine genome consensus sequence and breadth of coverage. The BAM files from mapping were used to generate comparative coverage plots using Integrative Genomics Viewer (v2.3.55) (15). The presence of candidate viral sequences found in VirCapSeq-VERT was investigated with specific PCR assays using primers and assay conditions shown in Table 1.

VirCapSeq-VERT yielded from 1.4 million to 62.2 million sequences per sample (average of 12.5 million sequences per sample). Together, RNA and DNA unbiased HTS yielded from 127.3 million to 391.0 million (average, 230.8 million) sequences per sample: 68.0 million to 134.7 million (average, 107.2 million) sequences per sample for RNA and 45.4 million to 312.9 million (average, 123.7 million) sequences per sample for DNA (Table 2).

**TABLE 2:** Viruses identified by two methods of high-throughput sequencing on plasma samples from Tanzanian patients with febrile illness

Sample	Unbiased HTS^a^					VirCapSeq-VERT					Confirmation (PCR result)
	Total no. of reads^b^	Virus(es) identified^c^	No. of viral reads	Genome coverage (%)	Terminal NCR coverage (%)^d^	Total no. of reads	Virus(es) identified	No. of viral reads	Genome coverage (%)	Terminal NCR coverage (%)	
Sample 1	**303,201,174**	DV2	1,032,128	100	100	**58,001,148**	DV2	54,739,618	99.9	99.4	Positive
Sample 2	**191,759,852**	DV2	616,291	100	100	**4,359,471**	DV2	1,663,516	99.9	98.0	Positive
		HPgV	27,953	95.8	100		HPgV	33,505	89.2	73.6	Positive
Sample 3	**240,702,142**	WNV	483	84.9	55.8	**2,186,326**	WNV	529	69.6	0.0	Positive
Sample 4	**233,216,250**	WNV	48	17.2	26.6	**1,413,970**	WNV	45	6.5	0.0	Positive
Sample 5	**156,590,622**	HIV-1	33,028	86.9	68.4	**62,168,103**	HIV-1	57,677,443	97.2	99.8	Positive
Sample 6	**317,810,172**	ND				**2,646,613**	ND				
Sample 7	**187,583,832**	HIV-1	138	39.2	0.0	**2,171,536**	HIV-1	4,586	80.7	49.0	Positive
		EBV	45	0.2	0.0		EBV	45	0.9	0.0	Positive
		HPgV	263	51.6	36.1		HPgV	2,935	59.7	69.6	Positive
Sample 8	**199,784,946**	ND				**2,601,036**	EBV	3	0.04	0.0	Positive
Sample 9	**206,125,028**	DV2	7,736	99.8	95.9	**2,807,056**	DV2	538,387	99.9	98.0	Positive
Sample 10	**391,029,362**	ND				**2,056,237**	ND				
Sample 11	**214,938,600**	ND				**1,958,631**	ND				
Sample 12	**127,295,376**	DV2	5,144,223	100	100	**8,104,818**	DV2	5,355,684	99.9	98.2	Positive

^a^ HTS, high-throughput sequencing.

^b^ The total number of reads is shown in boldface type for emphasis.

^c^ DV2, dengue virus 2; HPgV, human pegivirus; WBV, West Nile virus; ND, no virus detected; EBV, Epstein-Barr virus.

^d^ NCR, noncoding region.

Results were concordant across platforms. DV2 RNA was present in 4/12 samples, consistent with an outbreak of this virus in Dar es Salaam, Tanzania, during the period of sample collection (16). Human immunodeficiency virus type 1 (HIV-1) (2/12), WNV (2/12), HPgV (2/12), and Epstein-Barr virus (EBV) (2/12) were detected in the remaining plasma samples (Table 2). PCR confirmed the presence of nucleic acid from each of these viruses, including several low-titer samples. WNV (48 reads by unbiased HTS; 45 reads by VirCapSeq-VERT) was not detected by conventional PCR in sample 4; however, qPCR was positive (cycle threshold [*C*_*T*_] value of 32). HPgV was not detected by nested PCR in sample 7 but was positive by qPCR (*C*_*T*_ of 41). A weak qPCR result was obtained for EBV in sample 7 (45 reads by both sequencing methods; *C*_*T*_of 39). The detection of just three EBV reads by VirCapSeq-VERT in sample 8 was mirrored by a qPCR *C*_*T*_ of 40; EBV was not detected using unbiased HTS. A major advantage of both sequencing methods was the capacity to detect viral coinfections, as highlighted by the nearly complete genomes of DV2 and HPgV obtained from sample 2. We acknowledge that further investigation is required to determine the cause of fever in these patients, especially where multiple genomes were detected. The recovery of nucleic acid using HTS is insufficient for determining a causal link to disease; it is nonetheless valuable in defining potential viral candidates.

**FIG 1:**
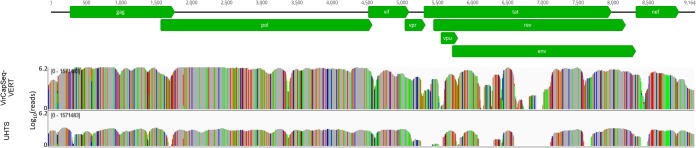
Read coverage plot for VirCapSeq-VERT and unbiased HTS for HIV-1 (GenBank accession number AY322190) in plasma sample 5. Colored lines indicate mismatches to the reference sequence (A [green], T [red], C [blue], and G [orange]). Sequencing depth is shown on the *y* axis in logarithmic scale. Nucleotide positions of the reference genome are indicated above each plot, and coding regions (light green) are marked with arrows. UHTS, ultrahigh-throughput sequencing.

Resource management is another important consideration in HTS. VirCapSeq-VERT obviates the need for sample preprocessing through DNase digestion or rRNA depletion to reduce the signal from host genetic material. It also increases sensitivity and reduces the complexity of bioinformatic analysis and computational power requirements. In this study, similar results were obtained using unbiased HTS with an average total of 230.8 million reads per sample and with VirCapSeq-VERT with an average of 12.5 million reads per sample. VirCapSeq-VERT, a method designed to capture coding sequence, had enhanced depth of coverage across viral coding regions (Fig. 1). Aside from HIV-1, and a low-titer HPgV in sample 7, unbiased HTS provided more complete coverage of the noncoding sequences at the termini of viral genomes that are essential for reverse genetics applications (Table 2). Unbiased HTS may also have advantages in virus discovery. In previous work, we demonstrated that VirCapSeq-VERT could not reliably detect viruses in which all potential probe hybridization sites vary by more than 40% (17). We have not encountered novel viruses with unbiased HTS that were missed with VirCapSeq-VERT in analysis of mammalian materials; however, tilapia lake virus, a virus that is decimating farmed tilapia worldwide, was discovered using unbiased HTS (18) and would not have been detected with the first release of VirCapSeq-VERT. The accuracy of data obtained by each sequencing method is highly dependent upon the reference viral database (19). The VirCapSeq-VERT probe set is updated annually to ensure coverage of new viruses; nonetheless, where VirCapSeq-VERT fails to yield a candidate viral pathogen or if bacterial or other parasitic agents have not been excluded, unbiased HTS should be considered.

HTS provides a comprehensive analysis of the plasma virome and is particularly well suited to situations where an infectious etiology has yet to be determined. Currently, the applicability of HTS in diagnostic medicine is limited by both cost and complexity of analysis. However, as costs decrease and analysis platforms improve, unbiased HTS and positive selection methods like VirCapSeq-VERT have the capacity to provide timely and important microbiological information that can help guide the response to an outbreak situation.
