# Erratum for Williams et al., “Investigation of the Plasma Virome from Cases of Unexplained Febrile Illness in Tanzania from 2013 to 2014: a Comparative Analysis between Unbiased and VirCapSeq-VERT High-Throughput Sequencing Approaches”

**DOI:** 10.1128/mSphere.00477-18

**Published:** 2018-09-19

**Authors:** Simon H. Williams, Samuel Cordey, Nishit Bhuva, Florian Laubscher, Mary-Anne Hartley, Noémie Boillat-Blanco, Zainab Mbarack, Josephine Samaka, Tarsis Mlaganile, Komal Jain, Valerie d’Acremont, Laurent Kaiser, W. Ian Lipkin

**Affiliations:** a Center for Infection and Immunity, Columbia University, New York, New York, USA; b Laboratory of Virology, University Hospitals of Geneva, Geneva, Switzerland; c University of Geneva Medical School, Geneva, Switzerland; d Department of Ambulatory Care and Community Medicine, University of Lausanne, Lausanne, Switzerland; e University of Lausanne Medical School, Lausanne, Switzerland; f Swiss Tropical and Public Health Institute, University of Basel, Lausanne, Switzerland; g Infectious Diseases Service, University Hospital of Lausanne, Lausanne, Switzerland; h Mwananyamala Hospital, Dar es Salaam, United Republic of Tanzania; i Ifakara Health Institute, Dar es Salaam, United Republic of Tanzania

## ERRATUM

Volume 3, no. 4, e00311-18, 2018, https://doi.org/10.1128/mSphere.00311-18. Page 4 of the PDF, Fig. 1 legend: in the third line of the legend, “coding regions” should be replaced with “genes.”

